# Multiple epithelioid angiosarcoma of stomach and small intestine with multiple lymph node metastases: A case report

**DOI:** 10.1097/MD.0000000000034024

**Published:** 2023-06-23

**Authors:** Jun-Hua Yu, Lu-Lu Cao, Jun Qian

**Affiliations:** a Department of Gastrointestinal Surgery, The Quzhou Affiliated Hospital of Wenzhou Medical University, Quzhou People’s Hospital, Quzhou, Zhejiang Province, China; b Department of Pathology, The Quzhou Affiliated Hospital of Wenzhou Medical University, Quzhou People’s Hospital, Quzhou, Zhejiang Province, China.

**Keywords:** case report, epithelioid angiosarcoma, gastrointestinal, lymph node metastasis, prognosis

## Abstract

Angiosarcoma is a mesenchymal soft tissue sarcoma with a tendency for vascular endothelial differentiation. It is highly malignant with a poor prognosis but has a low incidence. Epithelioid angiosarcoma of the gastrointestinal tract is rare, and simultaneous multiple lesions of the stomach and small intestine are even rarer. It is easy to be misdiagnosed clinically. We report on a case of preoperative misdiagnosis of gastric cancer and postoperative diagnosis of epithelioid angiosarcoma with multiple lymph node metastases.

**Patient concerns:**

A 75-year-old patient who was admitted to the hospital because of fatigue, melena and dysuria for >1 month.

**Diagnosis, interventions and outcomes:**

Gastroscopy revealed gastric fundus ulcer and the biopsy revealed poorly differentiated adenocarcinoma of the fundus. We performed a radical gastrectomy for gastric cancer during which multiple ulcers were found in the jejunum and resected. Postoperative pathology showed multiple epithelioid angiosarcoma in the stomach and small intestine with lymph node metastases. The patient did not receive further treatment and died 2 month after the surgery.

**Lessons:**

Gastrointestinal epithelioid angiosarcoma is one of the differential diagnoses of gastrointestinal adenocarcinoma and surgery is the main treatment. The lymph nodes are one of the main sites of metastasis.

## 1. Introduction

Angiosarcoma is a mesenchymal soft tissue sarcoma with a tendency for vascular endothelial differentiation. It is highly malignant, has a poor prognosis and accounts for 1% to 2% of all soft tissue tumors.^[1]^ As a subtype of angiosarcoma, epithelioid angiosarcoma was first reported by Weiss in 1982.^[2]^ It occurs in the skin, thyroid, kidney and breast.^[2–4]^ The gastrointestinal tract is an extremely rare site for angiosarcoma,^[5]^ which mainly occurs in the small intestine, and less in the stomach. Simultaneous multiple lesions in the stomach and small intestine are rare. Five cases of primary gastric lesions and 1 case of multiple lesions in the stomach and small intestine have been reported.^[6–10]^ Because of the low incidence of gastrointestinal epithelioid angiosarcoma, the pathogenesis has not been clarified, but it may be related to foreign bodies, exogenous toxins and long-term exposure to vinyl chloride.^[11]^ The disease can metastasize widely at early diagnosis, with poor healing and a median survival of 16 to 42 months.^[6]^ We present a case of multiple epithelioid angiosarcoma of the gastrointestinal tract with lymph node metastasis. This case was diagnosed preoperatively as gastric malignant tumor with upper gastrointestinal hemorrhage, and postoperatively by histopathological examination as gastric epithelioid malignant angiosarcoma. The lesion in the small intestine was found unexpectedly only when the jejunum was examined during esophagojejunostomy and was confirmed by histopathology. We describe the clinical manifestations, diagnostic process, immunohistochemical results, treatment and prognosis of this case to provide help for clinical diagnosis and treatment of gastrointestinal epithelioid angiosarcoma.

## 2. Case presentation

A 75-year-old male patient was admitted to the hospital because of fatigue, melena and dysuria for >1 month. He was diagnosed with upper gastrointestinal bleeding pending investigation. Symptoms started 1 month before presentation with recurrent melena and dysuria. The patient had no previous medical history. The patient had no family or personal history.

**Figure 1.**
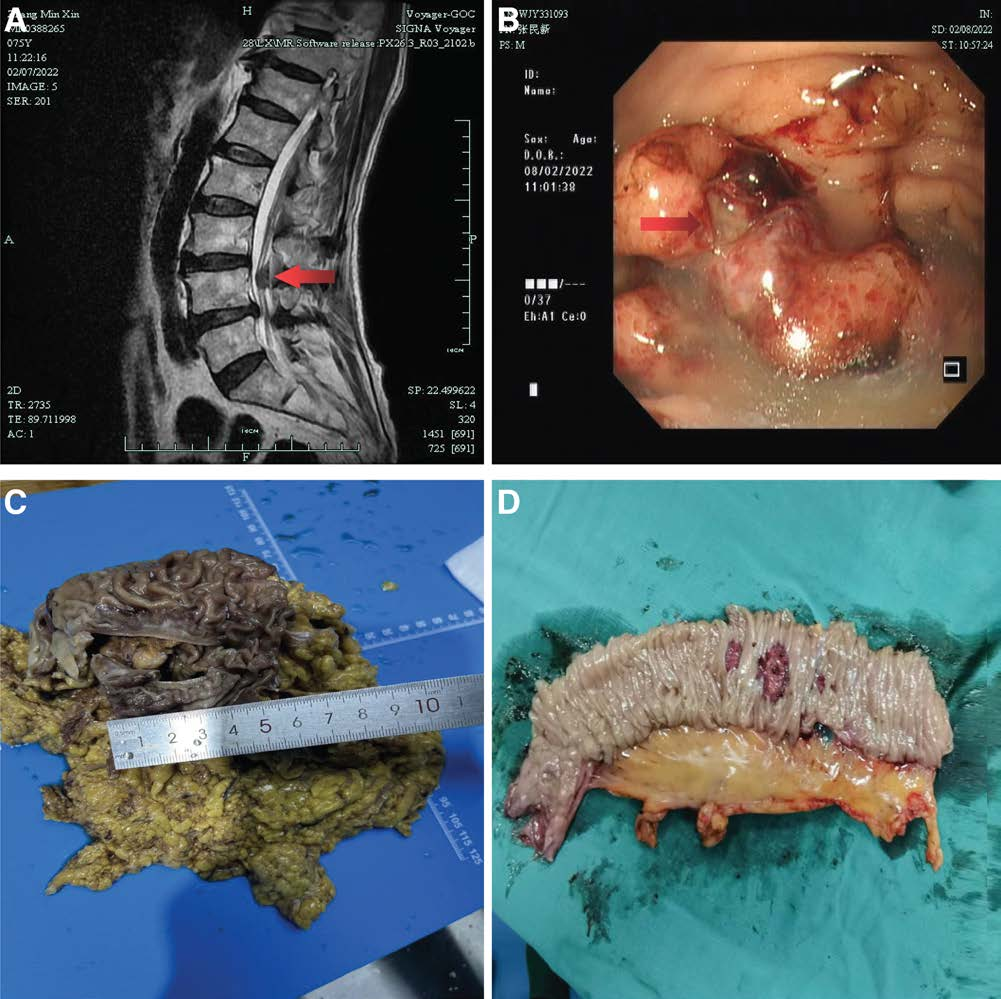
(A) Magnetic resonance imaging showing nodular abnormal signal shadows in the spinal canal at the level of the Lumbar 4 vertebrae (arrows). (B) Gastroscopy revealed an irregular ulcer of about 2.0 cm × 2.0 cm in the gastric fundus. (C) The total gastrectomy specimen showed an ulcer with a tumor size of 4 cm × 3 cm. (D) Small bowel resection specimen showed multiple ulcerated masses were seen, ranging in size from 0.7 cm to 2.

Physical examination on admission showed severe anemic appearance, pale conjunctiva (++), yellow sclera (−), soft abdomen, no tenderness, rebound pain, no mass. Laboratory tests showed hemoglobin 81 g/L, White blood cell 10.4 × 10^9^/L, C-reactive protein 29.6 mg/L, albumin 33 g/L, total bilirubin 9.5 μm/L, urea 6.72 mm/L, creatinine 72.2 μm/L, carcinoembryonic antigen 2.33 ng/mL, carbohydrate antigen 199 5.2 U/mL, carbohydrate antigen 724 2.11 U/mL, and positive fecal occult blood test (+++). Contrast-enhanced computed tomography (CT) of the whole abdomen showed no obvious abnormalities and CT of the chest did not demonstrate any metastasis. Magnetic resonance imaging of the lumbar spine showed abnormal nodular signal shadow in the spinal canal at the L4 vertebrae, which was suggestive of a possible neurogenic tumor (neurofibroma) (Fig. 1A). Gastroscopy revealed an irregular mass in the fundus of the stomach, approximately 2.0 cm × 2.0 cm in size, with a hard texture (Fig. 1B).

After a multidisciplinary discussion in our hospital, the patient was diagnosed with advanced gastric cancer with gastrointestinal bleeding (preoperative cT2-3N1-2M0) and primary spinal canal tumor with a progressive decrease of hemochrome after admission, the minimum being 64 g/L.

Comprehensive treatment based on surgery was recommended. Laparoscopic total gastrectomy and esophagojejunostomy were performed after surgical indications were assessed. Multiple nodular lesions, ranging in size from 0.5 cm to 2.0 cm, were found in the jejunum during intraoperative esophagojejunostomy, and an additional partial jejunectomy was performed (Fig. 1C and D).

**Figure 2.**
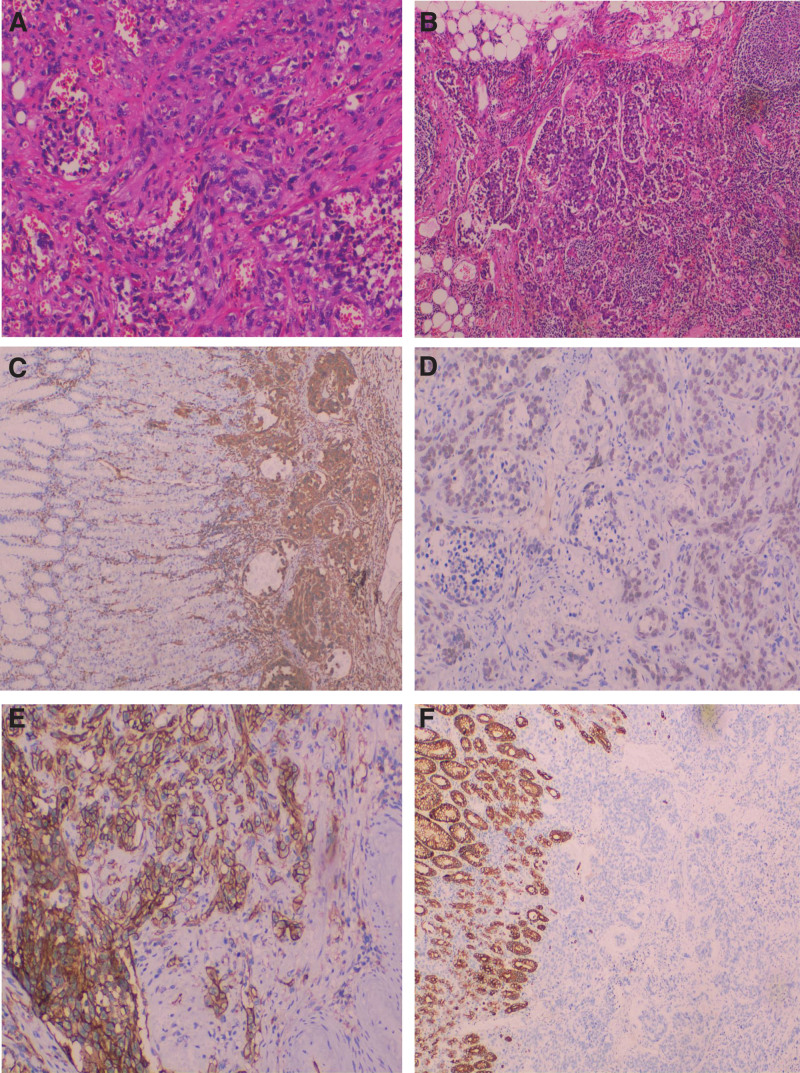
(A and B) Hematoxylin and eosin (H&E) staining show slit-like blood vessels, the tumor cells with large nuclei (A), and neoplastic blood vessels with obvious nucleoli were found in lymph nodes (B). (C–F) Immunohistochemistry analyses show tumor cells with Vimentin positivity (C), ERG positivity (D), CD31positivity (E), and CK positivity (F). All images are 100× magnification.

Postoperative pathology showed gastric fundus ulcerative epithelioid angiosarcoma, infiltration into deep muscle layers, negative resection margin, and lymph node metastasis (10/25), including pericardial (1/4), lesser curvature (9/16), subpyloric (0/1), greater curvature (0/2), group 8 (0/1), and group 10 (0/1). In ulcerative epithelioid angiosarcoma of the small intestine, the tumor size was 2 cm × 1.6 cm × 0.7 cm. There was infiltration into the subserosa membrane, neurovascular involvement, and peri-intestinal lymph node metastasis in 8/14. The postoperative Tumor Node Metastasis staging was considered as T2N3bMx. Immunohistochemical results showed: CK7 (+), CK20 (−), CDX-2 (−) and CEA (−) and B- Catenin (+), Her-2 (0), MLH-1 (+), MSH-2 (+), MSH-6 (+), PMS-2 (+), P53 (+), Ki-67 (40%). SMA (−), DOG-1 (−), CD34 (+), CD117 (−), LCA (−), CD31 (+), ERG (+), and Vimentin (+) (Fig. 2).

The patient was not treated further and died 2 mo later due to extensive metastasis.

## 3. Discussion and conclusions

The clinical manifestations of epithelioid malignant angiosarcoma of the digestive tract are nonspecific, and may include digestive bleeding, intestinal obstruction, perforation, and abdominal pain. Preoperative diagnosis is difficult; therefore, final diagnosis depends on histopathological examination and immunohistochemical staining. However, it is often differentiated from poorly differentiated adenocarcinoma, gastrointestinal stromal tumor, leiomyosarcoma, Kaposi sarcoma, metastatic tumor, and epithelioid hemangioendothelioma. Microscopically, multiple blood vessels can be seen communicating with each other, and the cells are distributed in sheets, clusters and nests. Large nuclei, obvious nucleoli, and vacuoles are the characteristic manifestations of epithelioid angiosarcoma.^[12]^ Because its histological morphology is similar to that of poorly differentiated carcinoma, it is easy to be misdiagnosed because of the lack of preoperative endoscopic biopsy samples.^[2,13]^ This case was also misdiagnosed as poorly differentiated gastric adenocarcinoma by preoperative pathology. Epithelioid angiosarcoma can express endothelial cell related markers ERG, CK, CK7, CD31, CD34 and Factor VIII–related antigen on immunohistochemistry. Immunohistochemical staining of this case showed CD31 (+), ERG (+), Vimentin (+), CD34 (+) and CK7 (+), which was consistent with previous reports.^[6]^

The rarity of this disease means that there is no accepted treatment. Surgical resection is the only possible treatment for gastrointestinal epithelioid angiosarcoma. Radiotherapy is usually used as an adjuvant therapy after non-radical resection to reduce the postoperative recurrence rate. Chemotherapy can be used as the main treatment for unresectable tumors or metastatic tumors, and drugs such as paclitaxel and epirubicin can be used.^[14]^ In recent years, programmed death-1 and vascular endothelial growth factor inhibitors have also been used, such as pembrolizumab, bevacizumab and sorafenib.^[14–16]^ In this case, total gastrectomy, partial resection of the small intestine, and lymph node dissection were performed. Postoperative histopathological examination revealed lymph node metastasis around the stomach (10/25) and lymph node metastasis in the small intestine (8/14), suggesting that lymph node dissection should be performed according to the surgical method for gastrointestinal cancer.

Dong et al^[12]^ reported cases of primary metastasis from lungs, liver and lower limbs to the small intestine, showing that hemorrhagic metastasis and lymph node metastasis are also common for epithelioid angiosarcoma. Lemus et al^[17]^ presented a case of an elderly patient who developed angiosarcoma causing spinal cord compression. In this case, for family economic reasons, there was a suspicious nerve fiber tumor in the spinal canal before surgery, which was still not identified postoperatively. However, metastasis from gastrointestinal epithelioid angiosarcoma was considered first, so it was not further treated.

The high degree of malignancy of angiosarcoma means that it is easy to relapse after surgery, resulting in poor overall treatment efficacy and poor prognosis. The present patient died 2 months after surgery. It has been reported that the 5-year survival rate in angiosarcoma patients was 31% to 43%, and the median survival time was 16 to 42 months.^[9,18,19]^

Gastrointestinal epithelioid angiosarcoma is one of the differential diagnoses of gastrointestinal adenocarcinoma. It is easy to be misdiagnosed clinically, leading to improper treatment. Surgery is an effective curative treatment. The lymph nodes are one of the sites of gastrointestinal epithelioid angiosarcoma metastasis, so lymph node dissection should be performed according to the surgical method for gastrointestinal cancer.

## Author contributions

**Project administration:** Jun Qian.

Writing – original draft: Junhua Yu.

Writing – review & editing: Lulu Cao.
